# Identification of DNA Methylation and Transcriptomic Profiles Associated With Fruit Mealiness in *Prunus persica* (L.) Batsch

**DOI:** 10.3389/fpls.2021.684130

**Published:** 2021-06-10

**Authors:** Karin Rothkegel, Alonso Espinoza, Dayan Sanhueza, Victoria Lillo-Carmona, Aníbal Riveros, Reinaldo Campos-Vargas, Claudio Meneses

**Affiliations:** ^1^Facultad Ciencias de la Vida, Centro de Biotecnología Vegetal, Universidad Andrés Bello, Santiago, Chile; ^2^Departamento de Producción Agrícola, Facultad de Ciencias Agronómicas, Centro de Estudios Postcosecha, Universidad de Chile, Santiago, Chile; ^3^FONDAP Center for Genome Regulation, Santiago, Chile

**Keywords:** peach, chilling injury, Rosaceae, epigenetics, cytosine methylation

## Abstract

Peach (*Prunus persica*) fruits have a fast ripening process and a shelf-life of days, presenting a challenge for long-distance consuming markets. To prolong shelf-life, peach fruits are stored at low temperatures (0 to 7 °C) for at least two weeks, which can lead to the development of mealiness, a physiological disorder that reduces fruit quality and decreases consumer acceptance. Several studies have been made to understand this disorder, however, the molecular mechanisms underlying mealiness are not fully understood. Epigenetic factors, such as DNA methylation, modulate gene expression according to the genetic background and environmental conditions. In this sense, the aim of this work was to identify differentially methylated regions (DMRs) that could affect gene expression in contrasting individuals for mealiness. Peach flesh was studied at harvest time (E1 stage) and after cold storage (E3 stage) for 30 days. The distribution of DNA methylations within the eight chromosomes of *P. persica* showed higher methylation levels in pericentromeric regions and most differences between mealy and normal fruits were at Chr1, Chr4, and Chr8. Notably, differences in Chr4 co-localized with previous QTLs associated with mealiness. Additionally, the number of DMRs was higher in CHH cytosines of normal and mealy fruits at E3; however, most DMRs were attributed to mealy fruits from E1, increasing at E3. From RNA-Seq data, we observed that differentially expressed genes (DEGs) between normal and mealy fruits were associated with ethylene signaling, cell wall modification, lipid metabolism, oxidative stress and iron homeostasis. When integrating the annotation of DMRs and DEGs, we identified a *CYP450 82A* and an *UDP-ARABINOSE 4 EPIMERASE 1* gene that were downregulated and hypermethylated in mealy fruits, coinciding with the co-localization of a transposable element (TE). Altogether, this study indicates that genetic differences between tolerant and susceptible individuals is predominantly affecting epigenetic regulation over gene expression, which could contribute to a metabolic alteration from earlier stages of development, resulting in mealiness at later stages. Finally, this epigenetic mark should be further studied for the development of new molecular tools in support of breeding programs.

## Introduction

Peaches [*Prunus persica* (L.) Batsch] and nectarines [*Prunus persica* (L.) Batsch var. nectarina] are climacteric stone fruits that belong to the Rosaceae family. Once harvested, *P. persica* fruits ripen fast within days at room temperature in spring and summer. To slow down the ripening process and prolong postharvest life, a cold storage is usually used at 0 °C for at least two weeks. However, this method can trigger a physiological disorder known as chilling injury (CI), which is manifested during ripening (Lurie and Crisosto, 2005). The symptoms of CI include mealy texture, flesh browning and/or bleeding, and leatheriness. The main cause of mealiness has been attributed to the metabolism of cell wall pectins, where mealy fruits are associated with an altered pectin methylesterase (PME) and endo-polygalacturonase (PG) activity (Brummell et al., 2004). Concomitantly, it has been suggested that mealiness is caused by the accumulation of insoluble calcium-pectate gel complexes in the middle lamella, trapping free water (Ben-Arie and Lavee, 1971). Another reported effect of CI on *P. Persica* is a reduced cell-to-cell adhesion, resulting in cell separation instead of cell rupture when chewing (King et al., 1989). More recently, studies have also suggested an alteration in reactive oxygen species (ROS) metabolism, mitochondrial respiration, sugar catabolism, amino acid usage, proteomic reprogramming and the importance of jasmonic acid signals in reducing CI via ethylene and sugars (Kan et al., 2011; Monti et al., 2019; Zhao et al., 2021).

Mealiness is genetically influenced and is also related with maturity date, since early cultivars have a lower susceptibility than later cultivars (Crisosto et al., 1999). In 2015, Nuñez-Lillo et al. (2015) studied an F2 Venus × Venus population, detecting one QTLs (Quantitative Trait Loci) for mealiness on linkage group 4 (LG4), explaining 34% of phenotypic variation. Within those QTLs, nine candidate genes related to cell wall synthesis, ethylene signaling and cold stress were identified. Later in 2019, Nuñez-Lillo et al. (2019) studied an F1 O’ Henry x NR-053 population, identifying an additional QTL in LG5, and an endo-1,3-beta-glucosidase as one of the candidate genes associated with mealiness.

At the transcriptomic level, Pons et al. (2014) studied a population (Pop-DG) segregating for CI, indicating that the transcriptome of peach fruits was changing during cold storage and was concomitant with the development of CI, highlighting that genes associated with cold response were differentially regulated between sensitive and resistant individuals. Afterward, Pons et al. (2016), analyzed the transcript profiles of the same population, but with different cold storage treatments and subsequent ripening. The results showed that during cold storage, the ripening program involving ethylene, auxin, and cell wall metabolism became desynchronized in susceptible fruits. Another study using a tolerant “Oded” and a relatively tolerant “Hermoza” peach cultivars identified that genes related to oxidative stress and biosynthesis of metabolites with antioxidant activity were positively correlated with CI tolerance (Puig et al., 2015). Moreover, the results suggested that ethylene signaling is related to CI tolerance, but also that auxins may play a role.

To better understand the molecular mechanisms modulating gene expression during plant development and adaptation, epigenomic approaches are an important layer of information. Epigenetic modifications involve histone changes, non-coding RNAs and DNA methylation, which influence chromatin structure and accessibility to genetic information (Zhang et al., 2018). In particular, cytosine DNA methylation has a key role in development, imprinting, silencing of transposable elements, abiotic and biotic responses (Law and Jacobsen, 2010). DNA methylation can be present under three different cytosine contexts: CpG, CHG, and CHH, where the H can be C, T, or A. An example of changes in DNA methylation during fruit ripening is the Colorless non-ripening (*Cnr*) mutant in tomato (*Solanum lycopersicum*), where colorless fruits loss cell-to-cell adhesion and have a mealy pericarp (Manning et al., 2006). This natural mutation involves high levels of cytosine methylation in a 286-bp region upstream of an SBP-box transcription factor, inhibiting fruit ripening and gene expression. A more recent study in tomato also showed that cytosine demethylation by a DNA glycosylase *SIDML2/ROS1* is necessary for the activation of ripening-induced genes and inhibition of ripening-repressed genes, revealing a critical role for DNA methylation during fruit ripening (Lang et al., 2017). In addition, changes in DNA methylation can be further used for the identification of novel markers, which will consider the genetic background, together with changing environmental conditions, as recently studied in *Prunus* (Prudencio et al., 2018; Rothkegel et al., 2020). In this sense, molecular approaches associated with agronomical traits should be implemented together with classical genetics in order to support breeding programs (García-Gómez et al., 2021).

Mealiness is a well-studied disorder, however, the molecular mechanisms leading to this phenotype are not entirely known. In order to elucidate if changes in DNA methylation levels are involved with susceptibility to cold storage, the aim of this work was to identify differentially methylated regions (DMRs) that could affect gene expression in contrasting individuals for mealiness at harvest time and after cold storage. This work will contribute with the first epigenomic study on mealiness, novel DMRs and additional differentially expressed genes (DEGs) that could be further studied as potential markers to differentiate susceptible peach fruits from tolerant fruits at harvest time.

## Materials and Methods

### Plant Material and Phenotyping

A previously characterized F2 V × V population of 151 individuals [*Prunus persica* (L.) Batsch] was used for fruit quality characters obtained from the self-pollination variety “Venus” (Nuñez-Lillo et al., 2015; Lillo-Carmona et al., 2020). This population located at Institute of Agricultural Research (INIA), Rayentué, Región de O’Higgins, Chile (34°32′14′′ S, 70°83′44′′ W), corresponds to six-year-old trees grown on G × N rootstock that segregates for mealiness, soluble solids content and maturity date. These fruits are freestone, with yellow and melting flesh. For season 2016–2017, peach fruits were harvested and at least five fruits were evaluated considering one individual with tolerance to cold and another individual with susceptibility to develop mealiness as determined in previous seasons (2012, 2013, and 2014) by our laboratory (Nuñez-Lillo et al., 2015; Lillo-Carmona et al., 2020). Fruits were harvested (E1 stage) and stored at −80 °C considering flesh firmness of 53 N and 0.8–1.2 values (average of each peach cheek measurement) of chlorophyll absorbance index (*I*_*AD*_) with a portable Vis/NIR DA-Meter. Flesh firmness was determined using a Fruit Pressure Tester (Effigi, Alfonsine, Italy) on both peach cheeks.

**FIGURE 1:**
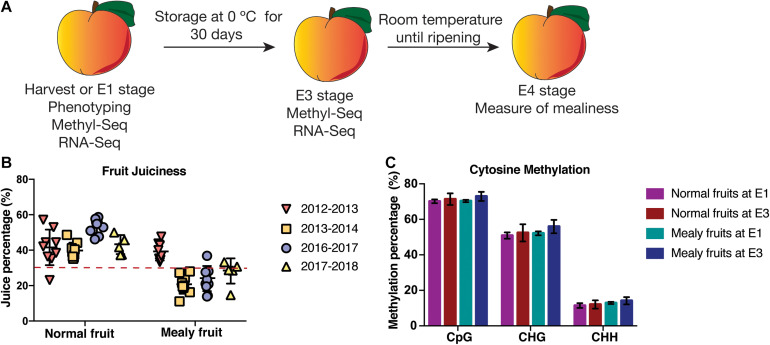
Phenotyping and global levels of DNA methylation in *P. persica* fruits susceptible and resistant to mealiness. **(A)** Experimental design for one resistant and one susceptible individual for mealiness. At harvest (E1), three fruits per tree were used for MethylC-Seq and RNA-Seq analysis, while another pool of fruits was stored in a cold chamber at 0 °C to promote mealiness. After 30 days of cold treatment (E3 stage), three fruits were used for MethylC-Seq and RNA-Seq analysis. At E4 stage, at least five fruits were ripened at room temperature and used for the measure of juice content. **(B)** Juice content (%) during four seasons in at least five fruits of an individual resistant and susceptible to mealiness, respectively. The dotted line represents a 30% threshold to consider fruits as mealy or normal. The same trees were evaluated in all seasons. **(C)** Absolute methylation levels (%) for cytosine contexts CpG, CHG and CHH (H = C, T or A) in normal and mealy fruits at E1 and E3.

A second pool of harvested fruits was stored at 0 °C for 30 days, corresponding to E3 stage (Figure 1A). After cold storage, fruits were stored at −80 °C until further use, while another group of fruits was stored at 20 °C for 7 days until flesh firmness was approximately 7 N, corresponding to E4 stage (ready to eat). Fruits from each pool and condition were evaluated for juiciness considering at least five biological replicates (Figure 1A). For E4 stage, firmness and juiciness were evaluated, while for E3 stage only firmness was measured. Juiciness was determined through a protocol described by Infante et al. (2009), where fruits with a value lower than 30% of juice were referred as mealy fruits, whereas fruits with higher values were considered as normal fruits. These juiciness values were compared using a mean separation test (ANOVA) and post-hoc Tukey-HSD test (*P* < 0.05). Finally, three fruits per individual (normal and mealy) were used at E1 and E3 stages for MethylC-Seq and RNA-Seq.

### MethylC-Seq

Bisulfite treatment was carried out from genomic DNA obtained from frozen peach flesh at harvest time (E1) and after cold storage (E3). Three fruits from a tolerant individual and additional three fruits from a sensitive individual were used as biological replicates. Genomic DNA was extracted using a DNeasy Plant mini kit (QIAGEN, Germantown, MD, United States) according to manufacturer’s instructions. The integrity of DNA was assessed in a 1.5% (p/v) agarose gel and concentration was determined with Qubit Fluorometer (Thermo Fisher Scientific, Waltham, MA, United States). Afterward, 100 ng of DNA was used for bisulfite treatment with the EZ DNA methylation gold kit (Zymo, Irvine, CA, United States) as previously described (Rothkegel et al., 2017). Twelve bisulfite treated libraries were generated considering two individuals (normal and mealy), two sampling points (E1 and E3) and three biological replicates. Libraries were generated with the TruSeq DNA Methylation kit (Illumina, San Diego, CA, United States) according to manufacturer’s instructions. All libraries were validated with Qubit Fluorometer and Fragment Analyzer (Advanced Analytical Technologies, Ankeny, IA, United States) and later sequenced in HiSeq 4000, 2 × 100 bp Paired-end mode (Illumina, San Diego, CA, United States). Raw data is available in NCBI sequence read archives (PRJNA705001).

### Processing of MethylC-Seq Reads and Identification of Differentially Methylated Regions

Initially, raw reads were filtered and adapters removed using Trim Galore^1^. Filtered reads were mapped against the reference genome of *P. persica* v.2.0.a1 using Bismark with no mismatches to obtain the methylation state of CpG, CHG, and CHH cytosine contexts (Krueger and Andrews, 2011; Verde et al., 2017). At the same time, reads were aligned to the plastid genome of *P. persica*, which is unmethylated, in order to obtain the bisulfite conversion rate. The bisulfite conversion rate was obtained by dividing the number of methylated cytosines and total cytosines of the chloroplast genome. For the identification of DMRs we used methylPipe considering cytosines covered with at least five reads and a *P*-value of 0.05 or less, a binsize of 10 and at least ten consecutives methylated cytosines using the find DMR function (Kishore et al., 2015). Comparisons were made for: (1) E3 vs. E1 in normal and mealy fruits; and (2) mealy vs. normal fruits at E1 and E3. A Kruskal-Wallis test was performed and regions with changes in methylation of at least two-fold were considered as DMR. Genes that overlapped with a DMR, including 2,000 bp upstream and downstream, were annotated as differentially methylated and used to perform a Gene Ontology (GO) analysis (FDR < 0.01) using AgriGO and REVIGO to decrease redundancy^2^.

### RNA-Seq

Total RNA from 4 gr of frozen peach mesocarp was isolated using the sodium borate decahydrate method described by Meisel et al. (2005). RNA integrity was assessed using Fragment Analyzer electrophoresis and Qubit Fluorometer. Twelve strand-specific libraries (2 individuals × 2 conditions × 3 biological replicates), were generated from 1 μg of total RNA using TruSeq Stranded mRNA kit (Illumina, San Diego, CA, United States). Validated libraries were sequenced in HiSeq 4000, 2 × 100 bp Paired-end mode and raw data is available in NCBI sequence read archive (PRJNA705416).

### Processing of RNA-Seq Reads and Identification of Differentially Expressed Genes

Sequenced reads were trimmed with Trim Galore and mapped to the reference genome of *P. persica* v.2.0.a1 using Spliced Transcripts Alignment to a Reference (STAR; Dobin et al., 2013). Uniquely mapped reads were normalized as trimmed mean of M-values (TMM) and used for DEG analysis with EdgeR considering a False Discovery Rate (FDR) < 0.01 and a fold-change of 1 or more (Robinson et al., 2010). Differentially expressed genes were obtained by comparing (1) E3 vs. E1 in normal and mealy fruits; and (2) mealy vs. normal fruits at E1 and E3. Variability in gene expression between mealy and normal fruits at E1 and E3 was considered to rank the top50 genes whose expression level was affected the most, represented as a heatmap. Additionally, DEGs that overlapped with DMRs, including 2,000 bp upstream and downstream of gene annotation, were considered as differentially methylated. Hypermethylated regions associated with downregulated genes and vice-versa were represented as heatmaps considering expression levels.

### Real Time qPCR Analysis

From total RNA, one microgram was treated with DNase I (Thermo Fisher Scientific), followed by the synthesis of cDNA using the SuperScript^TM^ first-strand synthesis system and oligo dT primers (Thermo Fisher Scientific), according to the standard protocol. Before use, each cDNA sample was diluted 1:10 with nuclease free water. The RT-qPCR assay was performed in an AriaMx real-time PCR system (Agilent Technologies, Santa Clara, CA, United States) with a master mix of KAPA SYBR^®^ FAST qPCR master mix (Kapa Biosystems, Wilmington, MA, United States), 10 μM of forward and reverse primers (Supplementary Table 1), ROX dye, template cDNA and PCR-grade water for a final volume of 10 μL. All RT-qPCR assays were performed using three biological and three technical replicates. The expression profiles of *PpeSAUR50* (Prupe.3G035000), *PpeERF4* (Prupe.4G176200), *PpeERF5* (Prupe.5G062000), and *PpeXET* (Prupe.3G172000) were normalized to *PpeTEF2* (EST TC3544) (Tong et al., 2009).

## Results

### Methylome Sequencing of Nectarine Fruits Contrasting for Mealiness

In order to determine the phenotypic parameters of mealiness, initially we used a V × V population of 151 siblings that was previously phenotyped during 2012, 2013, and 2014 for firmness, soluble solids contents, titratable acidity, weight and *I*_*AD*_ at E1 stage (Nuñez-Lillo et al., 2015). During these seasons, the average juiciness was 32.6%, ranging between 15 and 51% and obtaining a unimodal distribution. Afterward, during season 2016–2017, the same phenotypic parameters were measured, showing no significant differences for firmness, *I*_*AD*_ and soluble solids content (Lillo-Carmona et al., 2020). Moreover, juiciness ranged from 21,56% in mealy fruits, to 52.68% in normal fruits.

For the present study, we used the same two contrasting siblings from season 2016 to 2017 phenotyped by Lillo-Carmona et al. (2020) since they showed a consistent phenotype for mealiness during at least two seasons. For flesh juiciness, fruits showing a 30% or less of juice content were considered as mealy, obtaining fruits with a normal juice content (∼44.5%) during four seasons (2012–2013; 2013–2014; 2016–2017, and 2017–2018) and mealy fruits during three seasons (2013–2014; 2016–2017, and 2017–2018), showing a variable phenotype (Figure 1B).

Afterward, normal and mealy fruits were sequenced at E1 and E3 stages to analyze the methylation profiles that could be associated with the phenotype (Table 1). From total bisulfite reads, at least a 53.4% mapped uniquely to the *Prunus persica* v2.0 genome, whereas 17.2% reads showed no alignment. From uniquely mapped reads, approximately a 71.2% of cytosines were methylated in the CpG context, followed by a 52.8% in the CHG context and a 12.6% in the CHH context, with no significant differences between conditions (Figure 1C; Supplementary Table 2). At the same time, to analyze the bisulfite conversion rate, we mapped bisulfite reads to the chloroplast genome of peach, obtaining at least a 99.3% of conversion rate (Supplementary Table 3). To analyze the variability of global methylations, we conducted a PCA (Supplementary Figure 2). Despite PCA explains only a 25% of variability in DNA methylations, normal fruits at E1 and E3 are well separated from mealy fruits at the same conditions.

**TABLE 1:** Parameters of *Prunus persica* whole genome bisulfite sequencing and mapping of each sample and condition.

Sample	Sequence pairs analyzed	Uniquely mapped reads	Multiple mapped reads	Reads with no alignments	Genome coverage
Normal fruit at E1 R1	29,593,511	15,855,721	8,087,885	5,649,905	7X
Normal fruit at E1 R2	27,418,488	14,642,251	8,066,107	4,710,130	7X
Normal fruit at E1 R3	32,698,148	17,503,344	9,179,958	6,014,846	8X
Normal fruit at E3 R1	29,572,246	15,849,712	8,165,217	5,557,317	7X
Normal fruit at E3 R2	37,201,194	19,621,951	11,453,156	6,126,087	9X
Normal fruit at E3 R3	39,364,014	20,005,394	13,327,321	6,031,299	9X
Mealy fruit at E1 R1	30,242,762	15,788,636	8,575,159	5,878,967	7X
Mealy fruit at E1 R2	29,659,178	15,381,741	8,749,903	5,527,534	7X
Mealy fruit at E1 R3	32,770,016	15,350,153	11,278,650	6,141,213	7X
Mealy fruit at E3 R1	43,879,328	24,399,996	13,014,693	6,464,639	11X
Mealy fruit at E3 R2	36,227,923	19,872,745	10,317,302	6,037,876	9X
Mealy fruit at E3 R3	35,383,415	18,463,308	11,327,215	5,592,892	8X

*From the total sequence pairs analyzed, the number of uniquely mapped reads, multiple mapped reads and reads with no alignment was obtained. Genome coverage was estimated using a genome size of 225.7 Mb and considering only the uniquely mapped reads. Three biological replicates (R1, R2, and R3) were considered as three independent fruits per condition.*

**FIGURE 2:**
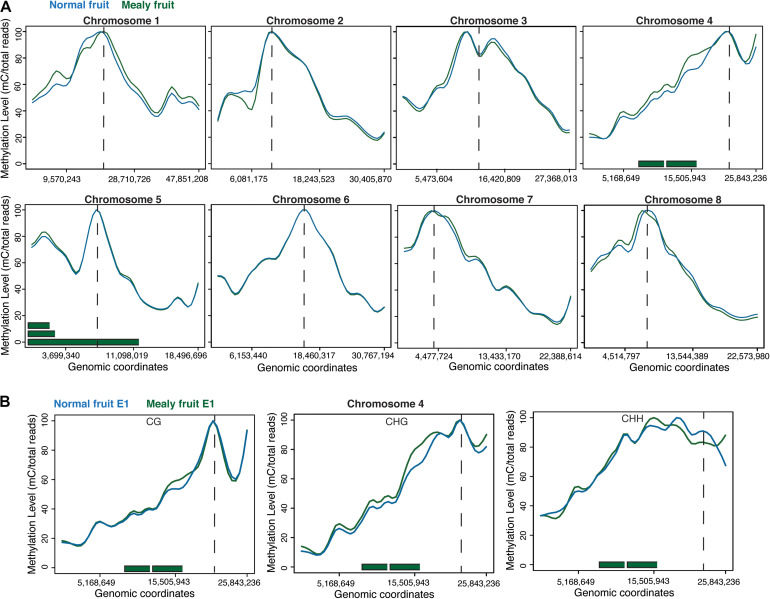
DNA methylation levels across the eight pseudomolecules of *P. Persica.* **(A)** Methylation levels considering all cytosine contexts in normal fruits (blue line) and mealy fruits (green line). Horizontal green bars represent the location of previously identified QTLs for mealiness, while dashed vertical lines indicates the approximate location of centromeres. **(B)** Methylation levels in CpG, CHG, and CHH contexts within chromosome 4, the main candidate associated with mealiness phenotype based on QTL studies.

When studying the methylation level within the eight chromosomes of peach, we observed that highest levels of methylation are associated with the average location of centromeres. Differences between mealy and normal fruits were present in Chr1 with increased methylation in mealy fruits and Chr2 with increased methylation at 6,081,175 bp in normal fruits. Additionally, Chr4 showed higher methylation levels in mealy fruits, whereas Chr8 possess regions with higher methylation in mealy fruits and others with higher methylation in normal fruits (Figure 2A). Furthermore, methylation differences within Chr4 were mostly contributed to CHG and CHH cytosine contexts, which also co-localized with previously reported QTLs for mealiness (Figure 2B) (Cantín et al., 2010; Nuñez-Lillo et al., 2015, 2019).

The candidate genes contained within these QTLs located in Chr4 and Chr5 are shown in Table 2. From these genes, those that presented DMRs between mealy and normal fruits at E1 are associated with gibberellin biosynthesis, with an increase of 45.9% in the methylation level of mealy fruits, signal transduction with an increase of 9.9%, protein transport with an increase of 4.9% and cell wall modification with a decrease of −13.7%. At E3, differences in methylation levels includes a gene related with carbohydrate transport, showing an increase of 46.6% in mealy fruits, genes related with protein phosphorylation (−24.8%) and iron ion binding (−10.8%) (Table 2).

**TABLE 2:** Genes that co-localized with QTLs for mealiness within chromosomes 4 and 5 and presented differentially methylated regions (DMRs) between mealy and normal fruits at E1 and E3 stages.

Stage	Gene ID	Description	Pathway	Methylation difference (%)	Location
E1	Prupe.4G128700	*ENT-KAURENE SYNTHASE*	Gibberellin biosynthesis	45.9	Intron
	Prupe.4G241500	*Mitochondrial import inner membrane translocase TIM50*	Protein transport	4.9	Downstream
	Prupe.5G074600	*Disease resistance protein-related*	Signal transduction	9.9	Upstream
	Prupe.5G069600	*PECTINESTERASE 68-RELATED*	Cell wall modification	−13.7	Intron
E3	Prupe.4G155700	*Bidirectional sugar transporter SWEET2*	Carbohydrate transport	46.6	Upstream
	Prupe.4G195300	*G-type lectin S-receptor-like serine*	Protein phosphorylation	−24.8	3′ UTR
	Prupe.5G077800	*CYTOCHROME P450-LIKE PROTEIN RELATED*	Iron ion binding	−10.8	Intron

*The methylation difference indicates an increase (positive) or decrease (negative) of methylation percentage in mealy fruits regarding to normal fruits.*

### Annotation and Identification of Differentially Methylated Regions in Normal and Mealy Fruits at E1 and E3 Stages

**FIGURE 3:**
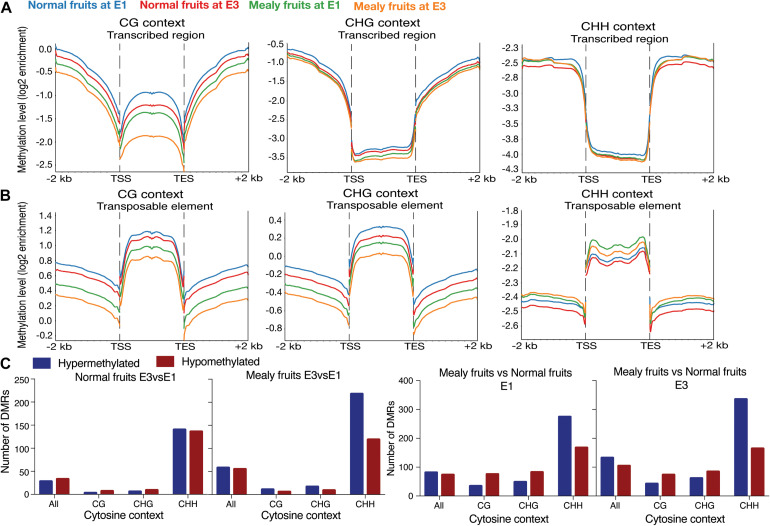
Average distribution of global DNA methylation throughout coding regions and number of differentially methylated regions. **(A)** DNA methylation level in annotated genes, including 2 kb upstream and downstream of the transcription start site (TSS) and transcription end site (TES), respectively. Each color represents a different condition. **(B)** DNA methylation level in annotated transposable elements (TE), including 2 kb upstream and downstream of TSS and TES. **(C)** Number of Differentially Methylated Regions (DMRs) close to annotated genes (up to 2 kb upstream and downstream) in normal fruits at E3 vs. E1 stages, mealy fruits at E3 vs. E1, mealy fruits vs. normal fruits at E1 and mealy fruits vs. normal fruits at E3. Blue bars indicate hypermethylated regions and red bars hypomethylated regions. DMRs where considered in all cytosine contexts and individual contexts (CpG, CHG, and CHH). Three biological replicates, a log2 enrichment difference >1 and a *p*-value < 0.05 were considered to identify a DMR.

To evaluate the distribution of methylations in all the annotated genes of *P. persica* (*n* = 26,873 genes), we obtained the methylation pattern within coding regions, including 2-kb upstream and 2-kb downstream (Figures 3A,B). For the CG context, methylation levels are higher upstream and downstream the transcribed regions, but with lower levels within TSS (transcription start site) and TES (transcription end site). Normal fruits showed a higher level of methylation than mealy fruits, however, all samples decreased their methylation when exposed to cold (E3 stage). On the contrary, for transposable elements (TE), CG methylations are higher within the TE rather than upstream and/or downstream. In the CHG context, methylation level is higher in upstream and downstream regions, maintaining low levels within transcribed regions. In the same context, TEs showed high levels of methylation, with a similar profile than CG methylations. In the CHH context, low levels of methylation are observed within the transcribed regions of genes, while high levels are present upstream and downstream. Regarding TEs, high levels of CHH methylation are observed within TE, but higher levels are present in mealy fruits at E1 and E3 stages when compared to normal fruits.

For the identification of DMRs, we considered the bisulfite conversion rate of each sample and a *p*-value < 0.05 for binominal test. When studying normal fruits at E3 vs. E1, we observed a similar amount of hypermethylated and hypomethylated regions, with most DMRs occurring in CHH context (Figure 3C). However, in mealy fruits at E3 vs. E1, CHH methylation showed higher number of hypermethylations (*n* = 218 DMRs) when compared to hypomethylations (*n* = 119 DMRs). When comparing mealy fruits vs. normal fruits at E1, we observed that differences in methylation mainly occurred in the CHH context with 274 hypermethylations and 167 hypomethylations in mealy fruits. Additionally, at E3, the number of hypermethylations in mealy fruits increased to 335 DMRs, while hypomethylations are maintained in 164 DMRs (Figure 3C). The annotation, methylation differences and description of each DMR in all cytosine contexts from normal fruits at E3 vs. E1, mealy fruits at E3 vs. E1, mealy fruits vs. normal fruits at E1 and E3 are detailed in Supplementary Tables 4–7, respectively.

**FIGURE 4:**
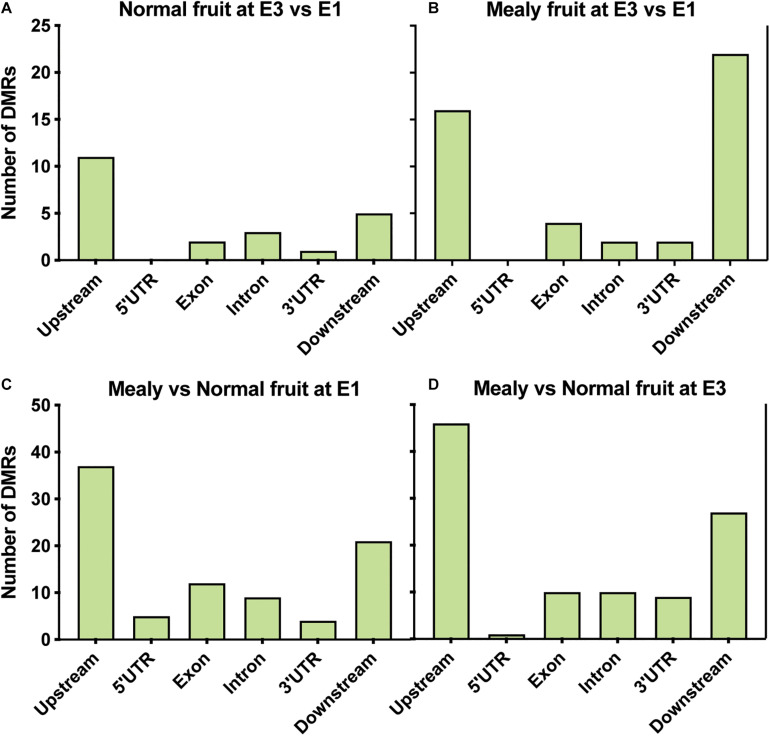
Location of DMRs within annotated genes of *P. persica*. Number of DMRs located upstream (up to 2 kb), 5’ UTR, exons, introns, 3′ UTR, and downstream (up to 2 kb) of annotated genes in samples from **(A)** normal fruits at E3 vs. E1 **(B)** mealy fruits at E3 vs. E1 **(C)** mealy vs. normal fruits at E1, and **(D)** mealy vs. normal fruits at E3.

From annotated DMRs, our interest was focused on which locations are more susceptible to change their methylation level. We observed that in normal fruits at E3, differences in methylation occurred upstream (*n* = 11 DMRs) of genes (Figure 4A), while in mealy fruits the number of DMRs is higher upstream (*n* = 16 DMRs) and downstream (*n* = 22 DMRs) of genes (Figure 4B). On the other hand, in mealy fruits vs. normal fruits, the number of DMRs located upstream of genes increased from 37 DMRs at E1, to 46 DMRs at E3 (Figures 4C,D).

To better understand which pathways may be affected by these changes in DNA methylation, we performed a GO analysis of the annotated DMRs between mealy and normal fruits (Table 3), where overrepresented molecular functions affected in mealy fruits corresponded to “ATP binding” and “purine nucleotide binding” at E1. At E3 stage, we observed an increase in overrepresented terms, where a higher frequency was obtained in “catalytic activity” with a 65.83%, “ATP binding” with 14.13% and “purine nucleotide binding” with 16.11%.

**TABLE 3:** Gene Ontology (GO) analysis of annotated genes associated with DMRs from mealy fruits vs. normal fruits at E1 and E3 stages.

Stage	Molecular function	Frequency	Term ID
E1	ATP binding	14.13%	GO:0005524
	Purine nucleotide binding	16.11%	GO:0017076
E3	Structural constituent of ribosome	2.68%	GO:0003735
	Catalytic activity	65.83%	GO:0003824
	Structural molecule activity	3.27%	GO:0005198
	ATP binding	14.13%	GO:0005524
	Ligase activity	3.54%	GO:0016874
	Purine nucleotide binding	16.11%	GO:0017076

*The columns show the overrepresented molecular functions, their frequency in each stage and the associated identity.*

### Transcriptome Sequencing and Identification of Differentially Expressed Genes in Normal and Mealy Fruits

In order to elucidate the transcriptional changes associated with the pattern of DNA methylations in response to chilling injury or mealiness, we analyzed RNA-Seq libraries from the same samples as MethylC-Seq (Table 4). Using samples from normal and mealy fruits at E1 and E3 stages, we obtained a 78–95% of uniquely mapped reads, 1.5–5.5% of reads that aligned in multiple regions and 1.5–19.6% of reads that did not align to the reference of *P. persica* genome v2.0. Considering only the reads that aligned uniquely, we followed further analysis.

**TABLE 4:** Parameters of *Prunus persica* transcriptome sequencing (RNA-Seq) and mapping of each sample and condition.

Sample	Processed reads	Uniquely mapped reads	Multiple mapped reads	Reads with no alignments	GC (%)
Normal fruit at E1 R1	26,432,188	22,634,900	703,271	3,094,017	47
Normal fruit at E1 R2	36,943,878	28,957,820	726,711	7,259,347	48
Normal fruit at E1 R3	32,478,417	26,966,410	635,225	4,876,782	48
Normal fruit at E3 R1	26,658,213	24,597,488	1,145,517	915,208	47
Normal fruit at E3 R2	20,609,623	19,335,278	840,540	433,805	46
Normal fruit at E3 R3	29,430,088	26,858,876	1,277,757	1,293,455	47
Mealy fruit at E1 R1	24,835,025	20,663,761	381,677	3,789,587	48
Mealy fruit at E1 R2	30,053,367	28,573,366	572,053	907,948	47
Mealy fruit at E1 R3	29,446,889	27,909,819	526,394	1,010,676	47
Mealy fruit at E3 R1	76,758,936	70,198,217	3,842,565	2,718,154	46
Mealy fruit at E3 R2	83,076,369	77,232,835	4,515,452	1,328,082	46
Mealy fruit at E3 R3	104,224,838	92,005,533	5,829,593	6,389,712	47

*Three biological replicates were considered as three independent fruits.*

**FIGURE 5:**
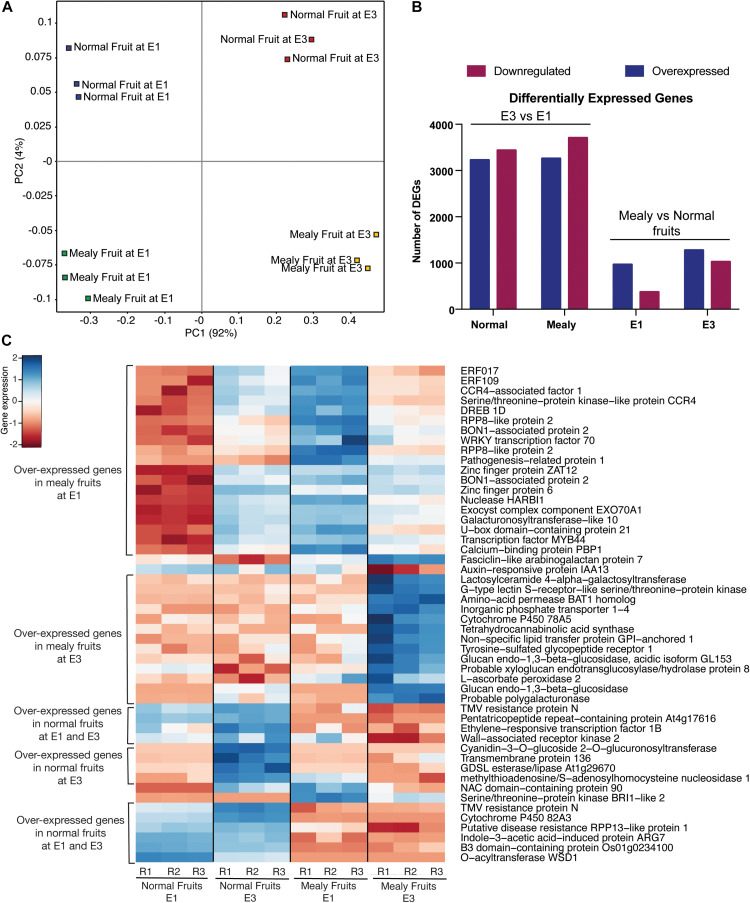
Transcriptomic profile of *P.persica* fruits susceptible and resistant to mealiness. **(A)** Principal Component Analysis (PCA) of global gene expression in normal (resistant) and susceptible fruits at E1 and E3. **(B)** Number of differentially expressed genes (DEGs) at E3 vs. E1, and in mealy vs. normal fruits. **(C)** Heatmap of the top 50 DEGs with higher variance in their transcript level between normal fruits and mealy fruits. Color key indicates the median log2 TMM-normalized values, where red to blue represents low to high levels of transcript, respectively.

To visualize the distribution of global transcriptomic data, we used a PCA (Figure 5A). In this case, all the conditions (normal fruit at E1, normal fruit at E3, mealy fruit at E1, and mealy fruit at E3) are well separated, while biological replicates grouped together, explaining a 92% of variability. For the identification of DEGs, we first considered normal and mealy fruits at E3 regarding to E1, and additionally compared mealy fruits vs. normal fruits at E1 and E3 (Figure 5B). For the first comparison, we obtained a higher number of downregulated genes (*n* = 3,436 DEGs) in normal fruits and in mealy fruits (*n* = 3,704 DEGs) at E3. However, when comparing mealy fruits vs. normal fruits, we observed a decreased number of DEGs, where most of them are overexpressed in mealy fruits at E1 (*n* = 966 DEGs), increasing toward E3 (*n* = 1,276 DEGs). The annotation and transcript level of each DEG is detailed in Supplementary Tables 8–11.

Afterward, we ranked the top 50 DEGs with higher variance between normal and mealy fruits (Figure 5C). Genes that showed an increased expression in mealy fruits at E1 includes *ETHYLENE-RESPONSIVE TRANSCRIPTION FACTOR*S (*ERFs*) 109 and 017, a cold acclimation gene *DREB1D*, defense genes *BON1* and *WRKY70*, and a cell wall gene *GALACTURONOSYLTRANSFERASE-like 10.* Another group of genes that showed an increased expression only in mealy fruits at E3 considers a lipid transport gene *LTPG1*, cell wall genes *XYLOGLUCAN XTH8* and a probable *POLYGALACTURONASE*, whereas a ROS scavenging gene *L-ASCORBATE PEROXIDASE APX2* was highly expressed both at E1 and E3 in mealy fruits. Genes that showed a decreased expression only in mealy fruits at E1 and E3 are *ERF1B*, a *CELL WALL-ASSOCIATED RECEPTOR KINASE 2 WAK2*, a *UDP-GLYCOSYLTRANSFERASE*, a *GDSL esterase/lipase* and a *O-ACYLTRANSFERASE WSD1* gene from the lipid metabolism, an *ARG7-like* gene associated with auxin and cold response, and a *CYTOCHROME P450 82A3* (*CYP82A3*) gene involved in sideretin biosynthesis in response to iron deficiency.

Additionally, in order to validate RNA-Seq data, we studied with real-time qPCR analysis the expression level of *PpeSAUR50*, *PpeERF5, PpeERF4*, and *PpeXET*, which were differentially expressed from RNA-Seq data between normal and mealy fruits (Supplementary Figure 2). The expression level of the four genes was similar between RNA-Seq and qPCR, showing differences between normal and mealy fruits.

### Integration of Methylomic and Transcriptomic Data

**FIGURE 6:**
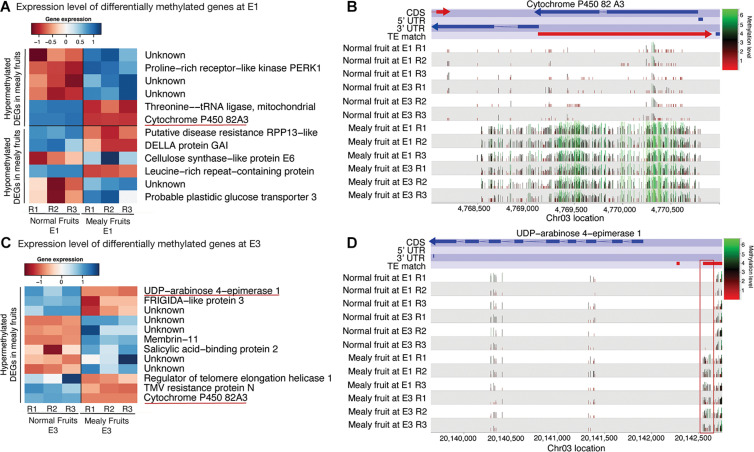
Differentially expressed genes between normal and mealy fruits associated with changes in DNA methylation. **(A)** Expression levels of DEGs overlapping with DMRs between normal and mealy fruits at E1. DEGs are grouped as hypermethylated and hypomethylated. **(B)** Cytosine methylation level of a hypermethylated region in mealy fruits compared to normal fruits. The DMR of approximately 2.500 bp is located within the coding region of a *CYP450* 82A3 (Prupe.3G066200) gene. Color key indicates methylation levels where green represents high levels and red, low levels. **(C)** Expression levels of DEGs overlapping with hypermethylated regions between normal and mealy fruits at E3. **(D)** DNA methylation level of a hypermethylated region in mealy fruits compared to normal fruits. The DMR of approximately 120 bp is located in the promoter region of an *UDP-ARABINOSE 4-EPIMERASE1* (Prupe.3G187800) gene.

For the integration of both methylation and transcriptomic data, we further studied only DMRs that co-localized with DEGs between normal and mealy fruits. At E1, hypermethylated genes that decreased their expression in mealy fruits correspond to a *THREONINE-tRNA LIGASE* and the recently mentioned *CYP82A3* (Figure 6A). On the contrary, hypomethylated genes that increased their expression in mealy fruits involves a *CELLULOSE SYNTHASE-LIKE PROTEIN E6* and a probable *GLUCOSE TRANSPORTER 3*. Regarding to *CYP82A3*, the gene viewer of DNA methylations in mealy fruits at E1 and E3 revealed an hypermethylation across the entire gene located within the anti-sense strand. Furthermore, this hypermethylation is associated with the silencing of *CYP82A3* and with the presence of a TE within the sense strand (Figure 6B). Additional hypermethylated genes at E3 that decreased their expression in mealy fruits includes an *UDP-ARABINOSE 4-EPIMERASE 1*, *FRIGIDA-like PROTEIN 3*, a *REGULATOR OF TELOMERE ELONGATION HELICASE 1*, and a *TMV RESISTANCE PROTEIN N* (Figure 6C). The methylation profile of *UDP-ARABINOSE 4-EPIMERASE 1* also shows an hypermethylation upstream the gene, only in mealy fruits, which co-localized with a TE that may affect this pattern (Figure 6D).

**FIGURE 7:**
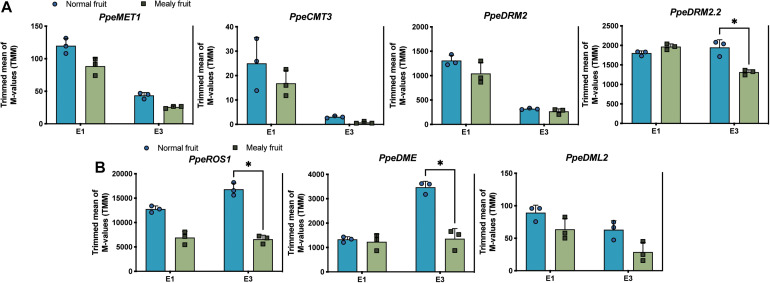
Expression levels of four putative DNA methyltransferases and three 5-mC glycosylases in normal and mealy *P.persica* fruits. Transcript levels of **(A)** methyltransferases *MET1* (Prupe.7G183100), *CMT3* (Prupe.6G011600), *DRM2* (Prupe.8G038800), and *DRM2.2* (Prupe.6G322700). **(B)** Transcript levels of 5-mC glycosylases *ROS1* (Prupe.7G118000), *DME* (Prupe.7G005000), and *DML2* (Prupe.6G119100). Transcripts were analyzed by RNA-Seq using three biological replicates. Asterisks represent significant differences (*p*-value ≤ 0.05) between normal and mealy fruits using a Two-Way ANOVA test. Error bars represent standard deviation (SD).

Regarding to the higher methylation levels observed in mealy fruits, we further wanted to know if DNA methyltransferases and DNA demethylases changed their expression level in fruits susceptible to mealiness. For this, we searched for *Arabidopsis thaliana* orthologous genes within the *P. Persica* genome. Four putative DNA methyltransferases were identified in peach, one *METHYLTRANSFERASE 1* (*MET1*), one *CHROMOMETHYLTRANSFERASE 3* (*CMT3*) and two *DOMAINS REARRANGED METHYLTRANSFERASE 2* (*DRM2*). Regarding to DNA demethylases, we identified one *REPRESSOR OF SILENCING 1* (*ROS1*), one *TRANSCRIPTIONAL ACTIVATOR DEMETER* (*DME*) and one *DEMETER-like 2* (*DML2*). The expression profile of each gene is shown in Figure 7. For the expression level of DNA methyltransferases, in general all of them decrease their expression after cold storage, however, only *DRM2.2* shows significant differences between mealy and normal fruits, where mealy fruits presented a lower expression of *DRM2.2* (Figure 7A). On the other hand, DNA demethylases *ROS1* and *DME* showed a significant downregulation in mealy fruits at E3 (Figure 7B).

## Discussion

### The Methylome of *P. persica* Fruits Reveals Epigenomic Differences Between Normal and Mealy Fruits Associated With Genetic Variance

Mealiness is a texture disorder characterized by the lack of juice from the flesh. In this study, we used samples from season 2016 to 2017, where no significant differences were obtained in firmness, *I*_*AD*_ and soluble solids (Lillo-Carmona et al., 2020). However, for fruit juiciness mealy fruits presented a 21.56% of juice and normal fruits a 52.68%, coincident with this textural disorder. Although mealiness is mostly influenced by the genetic background and maturation stage of the fruit, there is also an environmental factor that causes seasonal variability within the phenotype (Campos-Vargas et al., 2006), reason why a tree can produce mealy or normal fruits according to the season, as observed in Figure 1B. In fact, the contribution of the environmental effect over mealiness variance has been estimated between 36.5 and 57.5% in biparental populations of peach, coinciding with correlation values between seasons (Peace et al., 2005; Cantín et al., 2010; Nuñez-Lillo et al., 2015). Together with this, PCA results explained a 25% of variability in DNA methylations between normal and mealy fruits, indicating that most differences in global DNA methylation are due to genetic differences between siblings and to a lesser extend due to cold temperatures.

The methylome of nectarine fruits, obtained from two individuals with contrasting susceptibility to mealiness, was studied through MethylC-Seq before cold storage (E1 stage) and after 30 days at 0 °C (E3 stage). From methylome results, both in normal and mealy fruits, cytosine methylation was higher in the CpG context, followed by CHG and CHH, coinciding with previous methylome studies of *Arabidopsis thaliana*, apple (*Malus domestica borkh*.), *Populus euphratica* and sweet cherry (*Prunus avium* L.), showing that different pathways regulate DNA methylation (Lister et al., 2008; Daccord et al., 2017; Su et al., 2018; Rothkegel et al., 2020).

At the chromosome level, regions with higher methylation indicate the approximate location of centromeres, which are mainly comprised by TEs and repetitive elements (Verde et al., 2013). Therefore, methylation in all cytosine context is higher in pericentromeric heterochromatin (Lister et al., 2008). Additionally, differences in methylation between mealy and normal fruits within Chr1, Chr2, Ch4, and Chr8 could be attributed to the genetic variance and polymorphisms between individuals, affecting the methylation pattern. For instance, higher methylation levels within Chr4 of mealy fruits are close to pericentromeric heterochromatin and also co-localized with previously identified QTLs for mealiness, confirming genetic variability (Nuñez-Lillo et al., 2015).

Candidate genes for mealiness comprised within QTLs (Nuñez-Lillo et al., 2015; Nuñez-Lillo et al., 2019), also presented DMRs between mealy and normal fruits. At E1 stage, an increase in methylation of mealy fruits was observed in *ENT-KAURENE SYNTHASE*, a gene involved in gibberellin biosynthesis, a plant growth phytohormone suggested to reduce mealiness in nectarine fruits when applied exogenously (Lurie and Crisosto, 2005; Pegoraro et al., 2015). On the other hand, a gene showing a decrease of methylation in mealy fruits is *PECTINESTERASE 68*, a gene involved with cell wall metabolism. In this case, an alteration in the activity of polygalacturonases and pectinesterases can lead to the accumulation of gel-forming pectic compounds in the apoplast, retaining free water and resulting in mealy texture (Lurie et al., 2003; Brummell et al., 2004).

At E3 stage, the sugar transporter *SWEET2* presented increased methylation levels in mealy fruits, which could be associated with cold storage. Although the accumulation of sugars in peach fruits is an important trait for consumers, they also play an important role for cold tolerance by acting as osmolytes and protecting cell membranes (Ding et al., 2019).

### Distribution of Global DNA Methylation Patterns Is Related to Transposable Elements and Genetic Variance

The distribution and level of DNA methylation patterns are important factors for gene regulation and to avoid proliferation of TEs. For instance, gene body methylation is associated with CpG context and usually occurs within exons, but not in TSS and TES (Takuno and Gaut, 2013). According to the same study, methylated genes within their exons at CpG context, are constitutively expressed, however, CHG and CHH methylation tend to be lower within transcribed regions. Coincident with this, the methylation levels at CHG and CHH were lower within genes of peach, while methylation at CpG in transcribed regions was higher.

On the other hand, DNA methylation at gene promoters is associated with silencing by inhibiting the binding of transcription factors (Zhang et al., 2018). DNA methylation in non-coding regions can be consequence of nearby transposons and other repeats, which might be the case of peach, where methylation levels within TEs and neighbor regions is variable. Moreover, TEs respond to environmental stimuli. For example, in *A. thaliana* the repression of *VERNALIZATION INSENSITIVE 3* (*VIN3*) in the absence of cold is contributed by a TE located at the promoter region (Kim et al., 2010). In our study, the presence of TEs in mealy and normal fruits may generate a dynamic profile of DNA methylations between siblings, which in turn could alter gene regulation nearby. In fact, TEs represent a 29.6% of the peach genome, whereas a 7.54% of the genome are uncharacterized repeats (Verde et al., 2013).

The identification of DMRs between E3 and E1 stages in normal and mealy fruits showed that differences in the methylation level in response to cold storage occur mainly upstream and downstream of genes in CHH cytosines, being hypermethylated at E3 in both siblings, but with an increased number of DMRs in mealy fruits (Figure 3C). Several studies have indicated CHH methylation as a response to abiotic stress, suggesting a role for transient CHH methylation in response to environmental stimuli. For instance, during chilling accumulation in sweet cherry (*Prunus avium*), floral buds showed an increase in CHH methylation in genes associated with cold signaling, oxidation-reduction process, phenylpropanoid, and lipid metabolism (Rothkegel et al., 2020).

On the other hand, comparisons between mealy and normal fruits also showed hypermethylations of the CHH context in mealy fruits even before cold storage at E1, suggesting that genetic variance between siblings could result in increased DNA methylations in susceptible fruits. A GO analysis to identify which processes may be related with these DMRs in mealy fruits revealed molecular functions coincident with a metabolic response that is different in mealy fruits regarding to normal fruits. Regarding this, a previous proteomic analysis of peach fruits has showed that processes like ROS metabolism and cellular homeostasis are affected after cold storage (Nilo et al., 2010). In the same study, a decreased accumulation of catalases and ferritins was observed in cold stored fruits, suggesting an interaction between iron homeostasis and ROS levels.

### Genes Associated With Ethylene Signaling, Cell Wall Modification, Lipid Metabolism, Oxidative Stress, and Iron Homeostasis Are Differentially Expressed in Mealy Fruits

Responses to low temperatures involve transcriptomic and metabolic reprogramming. At the transcriptomic level, PCA and the number of DEGs showed that gene expression is affected not only by the genotype of each individual, but also by the fruit condition (E1 and E3). Moreover, most DEGs were obtained between E1 and E3 rather than in mealy vs. normal fruits as observed in methylome data. This difference in gene expression may be due to an energetic reprogramming in order to establish a new metabolic homeostasis for acclimation under cold conditions, which is regulated by several factors, such as signaling pathways and transcription factors (Fürtauer et al., 2019).

**FIGURE 8:**
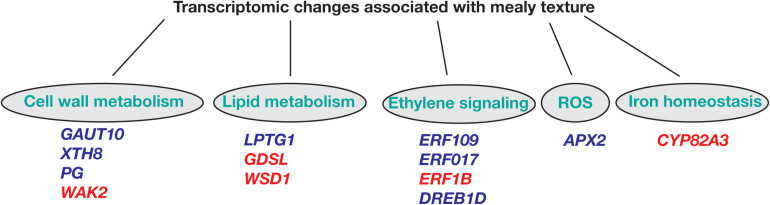
Hypothetical scheme of differentially expressed genes between mealy and normal fruits associated to metabolic pathways leading to mealiness in *P. persica* fruits. Biological processes of cell wall metabolism, lipid metabolism, ethylene signaling, ROS (reactive oxygen species) and iron homeostasis are represented. Genes highlighted in blue are overexpressed, while red colored genes are downregulated in mealy fruits. *GAUT10: GALACTURONOSYLTRANSFERASE-like 10; XTH8: XYLOGLUCAN ENDOTRANSGLUCOSYLASE/HYDROLASE PROTEIN 8; PG: POLYGALACTURONASE; WAK2: WALL-ASSOCIATED RECEPTOR KINASE 2; LPTG1: NON-SPECIFIC LIPID TRANSFER PROTEIN GPI-ANCHORED 1; GDSL esterase/lipase; WSD1: O-ACYLTRANSFERASE; ERF: ETHYLENE-RESPONSIVE FACTOR; DREB1D: DEHYDRATION-RESPONSIVE ELEMENT BINDING 1D; APX2: L-ASCORBATE PEROXIDASE 2; CYP82A3: CYTOCHROME P450 82 A3*.

Regarding overrepresented pathways, ethylene signaling is essential for a series of developmental processes, such as growth and senescence, and responses to abiotic and biotic stress. In peach, we identify the ethylene response factors *ERF109*, *ERF017*, and *DREB1D* with higher expression in mealy fruits at E1 stage when compared to E3, while *ERF1B* was downregulated at E1 and E3 (Figure 8), coincident with previous studies that suggested that CI is associated with a decreased ethylene production (Wang et al., 2017; Nilo-Poyanco et al., 2018).

Another determinant factor for the development of mealiness is the cell wall, where initial studies have found an association between mealiness and the altered activity of pectin methylesterases (PME) and endo-polygalacturonases during cold storage, resulting in higher levels of soluble pectins and mealy texture (Buescher and Furmanski, 1978; Ben-Arie and Sonego, 1980; Lurie et al., 1994). Concomitantly, in mealy fruits at E1 we identify a *GALACTURONOSYLTRANSFERASE-like 10* (*GAUT10*) showing higher expression (Figure 8). Afterward at E3, *XYLOGLUCAN XTH8* and a probable *POLYGALACTURONASE* (*PG*) presented an increased expression, while a *CELL WALL-ASSOCIATED RECEPTOR KINASE 2 WAK2* was downregulated, suggesting an altered cell wall metabolism during cold storage.

As one of the first barriers to the environment, the cell membrane is important to integrate the environmental signal into the cell. In the case of low temperatures, a decrease in membrane fluidity correlates with the proportion of desaturated fatty acids, affecting the metabolism of lipids (Martiniere et al., 2011). Moreover, in *A. thaliana*, the levels of polyunsaturated fatty acids are pivotal for survival to low temperatures (<12°C) (Miquel et al., 1993). In peach, we have found that *LTPG1*, associated with lipid transport, is overexpressed in mealy fruits after cold storage (Figure 8). While a *GDSL esterase/lipase* and a *O-ACYLTRANSFERASE WSD1* are downregulated in mealy fruits in both conditions, indicating that the lipid metabolism is differentially regulated probably due to the genetic background of each individual since these changes occurred before cold storage. In fact, a recent study using the same peach individuals has identified 119 metabolites and 189 lipids with significant differences in abundance between juicy and mealy fruits, where lipids were proposed as biomarkers for mealiness at E1 and E3 stages (Lillo-Carmona et al., 2020).

Regarding oxidative stress, the signaling of ROS is associated with biotic and abiotic responses. Usually, an abiotic stress like low temperatures increase the levels of ROS because of a lower stability of scavenging enzymes (Ding et al., 2019). Some of these scavenging enzymes correspond to superoxide dismutase, ascorbate peroxidase, catalase, and glutathione peroxidase (Müller and Munné-Bosch, 2015). In this study, we identify a *PEROXIDASE APX2* that is highly expressed in mealy fruits before and after cold storage (Figure 8), indicating that susceptible fruits might generate lower ROS levels and thus, a lower signal to induce a cold response.

The connection between ROS and iron homeostasis has also been reported, where hydrogen peroxide (H_2_O_2_) molecules react with Fe^2+^ to generate hydroxyl radicals, which attack pectin polysaccharides, decreasing mealiness susceptibility (Airianah et al., 2016; Choudhury et al., 2016). Furthermore, we have found that a *CYP82A3* gene associated with iron deficiency is silenced in mealy fruits (Figure 8) and thus, we hypothesize that low levels of Fe^2+^ due to *CYP82A3* silencing and low levels of ROS due to high *APX2* expression, could result in lower hydroxyl radicals, promoting a favorable environment for pectin accumulation and mealiness development.

### The Downregulation of *CYP82A3* and *UDP-ARABINOSE 4* in Mealy Fruits Is Accompanied by Higher Methylation Levels and the Presence of a TE

The superfamily of CYTOCHROME P450 has an important role in promoting plant growth, development, and stress responses with detoxification pathways (Xu et al., 2015). Regarding development, CYP 450s are involved in several metabolic pathways, such as cytokinin and flavonoid biosynthesis, and iron deficiency (Kruse et al., 2008; Berim and Gang, 2013). As previously mentioned, we have found that a putative *CYP82A3* is not expressed in mealy fruits, which is associated with its hypermethylation. Moreover, this hypermethylation coincides with the localization of a TE in the opposite strand, suggesting that the presence of the TE might be responsible for an increase in methylation levels, affecting gene expression.

Another downregulated gene in mealy fruits is *UDP-ARABINODE 4*. L-arabinose (Ara) in plants is an important component of glycoproteins, flavonoids, signaling peptides and cell wall polysaccharides, participating in cell wall biosynthesis (Rautengarten et al., 2017). In this sense, an imbalance of Ara affects directly the composition of the cell wall and may also be involved in mealiness texture, where a decreased cell-to-cell adhesion causes cell separation instead of cell rupture during chewing (Brummell et al., 2004). In our study, the downregulation of *UDP-ARABINOSE 4 EPIMERASE 1* is associated with an increase in the methylation level upstream. The encoded protein catalyzes the 4-epimerization of UDP-_*D*_-Xyl to UDP-_*L*_-Ara (Burget et al., 2003), suggesting that the levels of xylose could also be affected in mealy fruits. Interestingly, this hypermethylation co-localized with a TE match, which may affect gene expression in a similar way than *CYP82A3*. Altogether, these results highlight that genetic variability due to TEs and repetitive elements results in increased DNA methylation levels in mealy fruits before cold storage, which could affect gene expression and tolerance during cold storage.

### DNA Glycosylases Are Downregulated in Mealy Fruits, Contributing With an Increase in DNA Methylations

DNA methyltransferases and glycosylases are key factors for the establishment and maintenance of methylation patterns. Methylation in the CpG context is the most abundant and is maintained during DNA replication by DNA METHYLTRANSFERASE 1 (MET1) (Finnegan et al., 1996). While methylation in the CHG context is maintained by a reinforcing loop involving CHROMOMETHYLASE 3 (CMT3) and histone marks (H3K9) (Du et al., 2012). Methylation in CHH occurs *de novo* and is maintained by DOMAINS REARRANGED METHYLTRANSFERASE 2 (DRM2), together with non-coding RNAs in a pathway known as RNA-directed DNA Methylation (RdDM) (Law and Jacobsen, 2010). In peach we have found four homologous to these DNA methyltransferases, *PpeMET1*, *PpeCMT3*, *PpeDRM2*, and *PpeDRM2.2*. The transcript levels for all these genes have shown a decrease in expression at E3 stage in normal and mealy fruits, indicating that the regulation of these genes is affected by low temperatures in peach fruits.

On the other hand, DNA demethylation involves the participation of glycosylases REPRESSOR OF SILENCING 1 (ROS1), DEMETER (DME) and DEMETER-like (DML), which act during DNA reparation through a process of base excision, removing methylated cytosines (Gong et al., 2002; Choi et al., 2002; Ortega-Galisteo et al., 2008). In peach, we have identified *PpeROS1, PpeDME*, and *PpeDML2*, whose expression increased after cold storage only in normal fruits, indicating a higher control of DNA methylations. On the contrary, mealy fruits were associated with higher levels of DNA methylations, possibly due to a lower expression of DNA glycosylases. These results are concomitant with the key role described for DNA demethylation during fruit ripening by Lang et al. (2017), where mutant tomato plants for a *ROS1* homolog gene failed to ripen because ripening genes were silenced by DNA methylations.

## Conclusion

In conclusion, in this study we obtained different DNA methylation patterns between normal and mealy fruits, showing higher methylation levels in mealy fruits, particularly in the CHH context. Moreover, these differences in methylation appears to be mostly influenced by genetic differences between siblings (e.g., TEs and repetitive elements), coinciding with previous QTL studies. When integrating RNA-Seq data, we found that *CYP82A3* and *UDP-ARABINOSE 4 EPIMERASE 1* are downregulated in mealy fruits, coinciding with the presence of TEs, possibly affecting iron homeostasis and cell wall metabolism. From gene expression data, a lower expression of DNA glycosylases during cold storage could contribute with higher methylation levels in mealy fruits. Taken together, this study provides an additional layer of information to better understand the complex phenotype of mealiness. Moreover, the patterns of DNA methylation and expression levels of *CYP82A3* and *UDP-ARABINOSE 4* could be further studied as novel markers for their use, together with molecular markers, in order to support peach breeding programs.

## Data Availability Statement

The datasets presented in this study can be found in online repositories. The names of the repository/repositories and accession number(s) can be found below: https://www.ncbi.nlm.nih.gov/genbank/, PRJNA705001; PRJNA705416.

## Author Contributions

CM, RC-V, and KR designed the research. AE, AR, and VL-C conducted field work and phenotyping. KR generated MethylC-Seq libraries. AE and KR contributed with bioinformatic analysis of MethylC-Seq. DS contributed with RNA-seq and qPCR experiments, while AR, AE, and KR performed bioinformatic analysis of RNA-seq. CM conducted statistical analysis and KR wrote the original manuscript. KR, CM, VL-C, DS, and RC-V edited and reviewed the original manuscript. All authors read and approved the submitted version.

## Conflict of Interest

The authors declare that the research was conducted in the absence of any commercial or financial relationships that could be construed as a potential conflict of interest.

## Footnotes

1 https://www.bioinformatics.babraham.ac.uk/projects/trim_galore/

2 http://bioinfo.cau.edu.cn/agriGO/analysis.php
