# Supplementary material for: Identification of DNA Methylation and Transcriptomic Profiles Associated With Fruit Mealiness in Prunus persica (L.) Batsch
Source: Front Plant Sci. 2021 Jun 10;12:684130. doi: 10.3389/fpls.2021.684130 (PMC8222998; doi:10.3389/fpls.2021.684130)
Supplement: Supplementary file 2 [file Data_Sheet_2.docx]

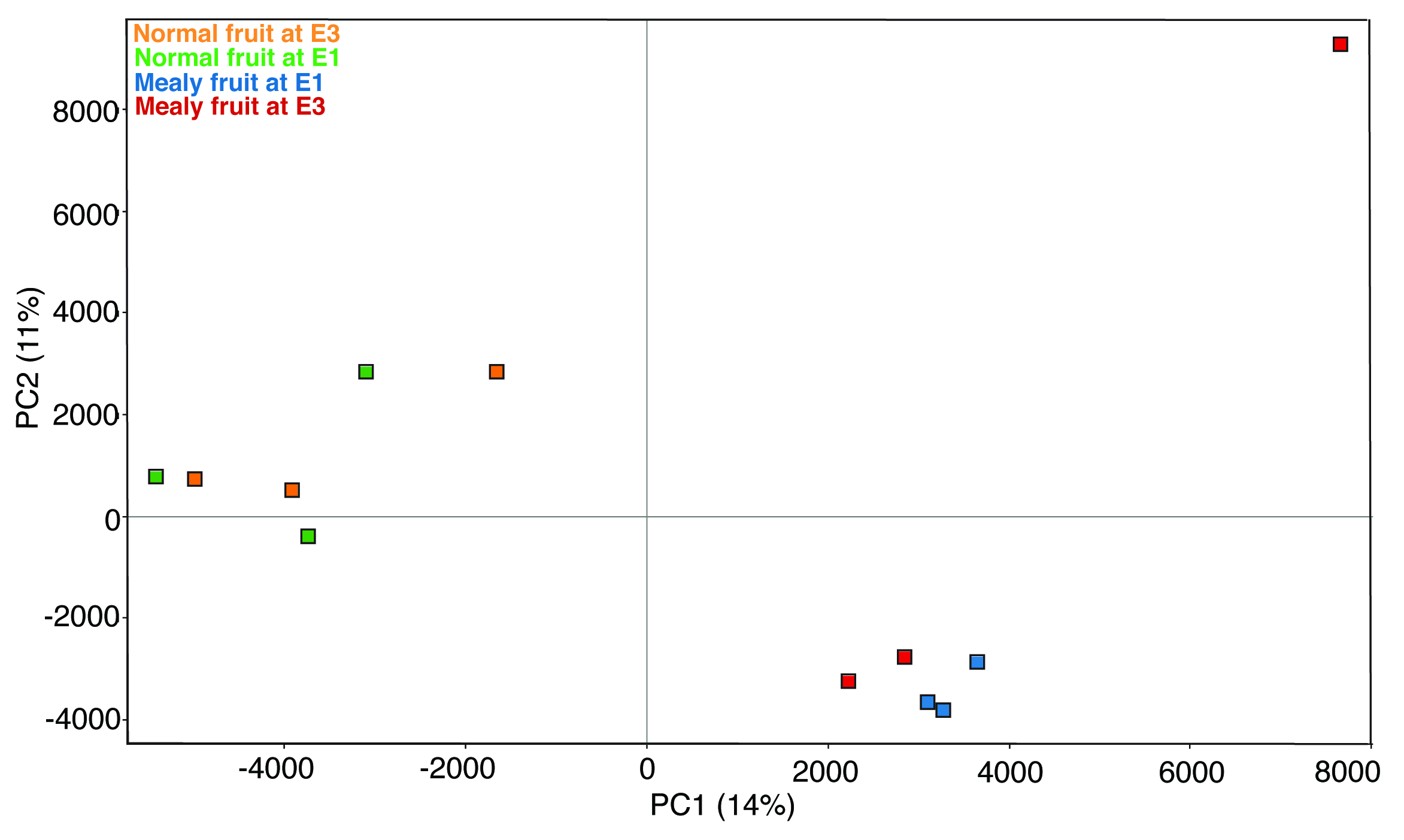


**Figure S1.** Principal Component Analysis (PCA) of global DNA methylations in *P. persica* fruits. The analysis was performed considering all methylated cytosines from normal and mealy fruits at E1 and E3 stages*.*


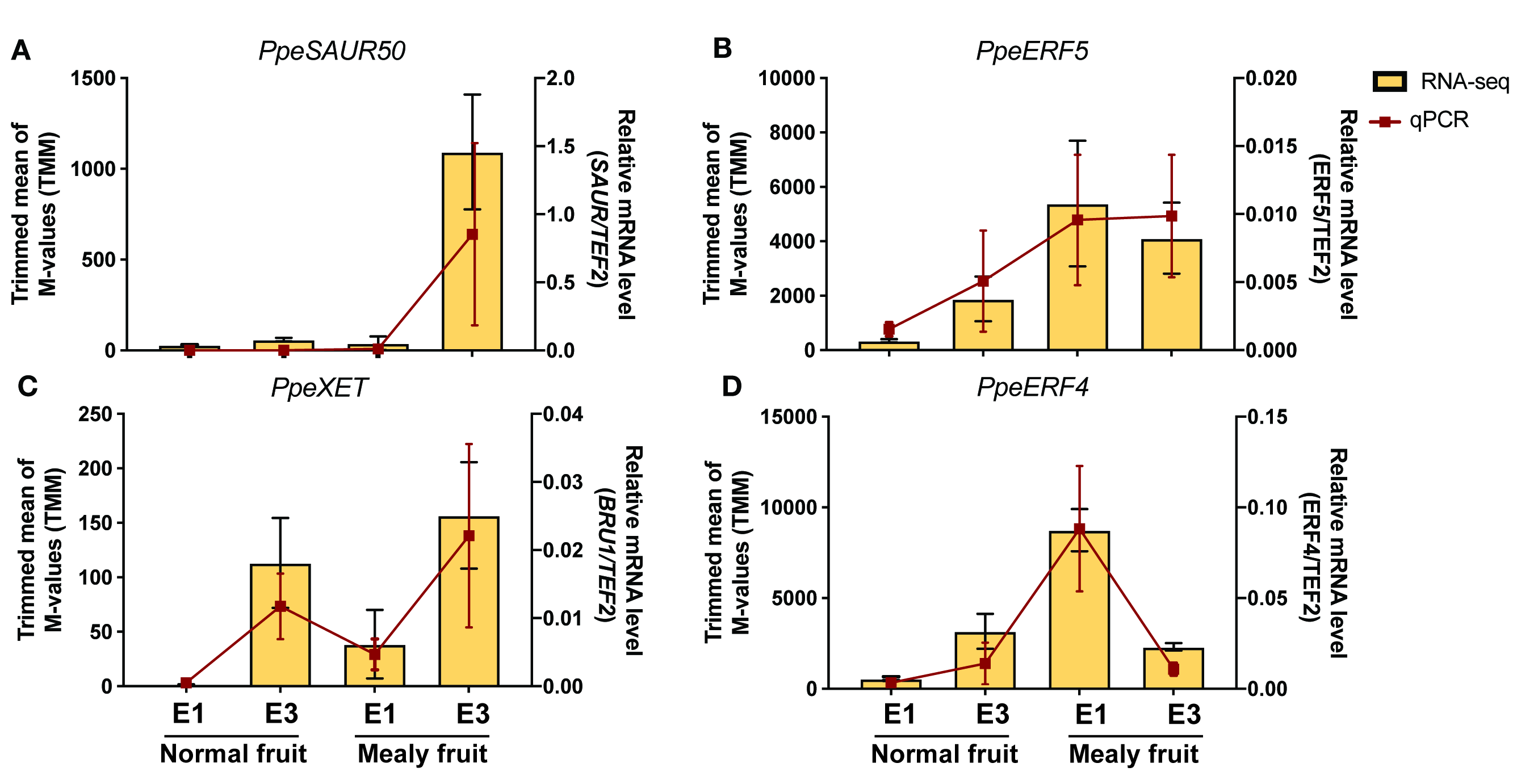


**Figure S2.** Expression levels of four differentially expressed genes between normal and mealy fruits, and between E1 and E3 stages. **(A)** Auxin responsive protein SAUR50 (Prupe.3G035000), **(B)** ethylene-responsive transcription factor *ERF5* (Prupe.5G062000) **(C)** Xyloglucanendohydrolase XET (Prupe.3G172000), **(D)** ethylene-responsive transcription factor *ERF4* (Prupe.4G176200)*.* Transcripts were analyzed by Real-Time qPCR using three biological and three technical replicates. Error bars represent standard deviation (SD).
